# Poly[μ-aqua-di-μ-benzoato-lead(II)]

**DOI:** 10.1107/S1600536809028542

**Published:** 2009-07-25

**Authors:** Jiantong Li, Juan Yang

**Affiliations:** a Department of Physical Chemistry, Henan Polytechnic University, Jiaozuo 454003, People’s Republic of China

## Abstract

The reaction of lead(II) nitrate and benzoic acid in aqueous solution yields the title polymer, [Pb(C_7_H_5_O_2_)_2_(H_2_O)]_*n*_. The asymmetric unit contains one Pb^II^ ion, two benzoate ligands and one water mol­ecule. The Pb—O bond distances are in the range 2.494 (4)–2.735 (4) Å. The Pb⋯Pb distance is 4.0683 (4) Å, indicating an insignificant metal–metal inter­action. The Pb^II^ atom has a distorted penta­gonal-bipyramidal geometry chelated by two carboxyl­ate O atoms. The Pb atoms are bridged through a coordinating water mol­ecule and two carboxyl­ate O atoms from another two benzoate ligands, giving an infinite three-dimensional supra­molecular structure. O—H⋯O hydrogen-bonding inter­actions involved the coordinating water and carboxyl­ate O atoms enhance the stability of the supra­molecular arrangement.

## Related literature

For general background to lead(II) compounds, see: Shi *et al.* (2007 ▶); Fan & Zhu (2006 ▶); Wang *et al.* (2006 ▶); Kim *et al.* (2001 ▶). For related structures, see: Shi *et al.* (2007 ▶).
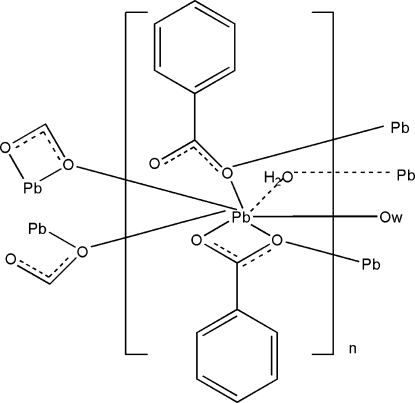


## Experimental

### 

#### Crystal data

[Pb(C_7_H_5_O_2_)_2_(H_2_O)]
*M* *_r_* = 467.43
Monoclinic, 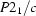

*a* = 15.4118 (12) Å
*b* = 7.5122 (6) Å
*c* = 11.4856 (9) Å
β = 91.2930 (10)°
*V* = 1329.42 (18) Å^3^
*Z* = 4
Mo *K*α radiation
μ = 12.71 mm^−1^
*T* = 295 K
0.40 × 0.10 × 0.08 mm

#### Data collection

Bruker APEXII CCD area-detector diffractometer
Absorption correction: multi-scan (*SADABS*; Bruker, 2007 ▶) *T*_min_ = 0.1, *T*_max_ = 0.247 (expected range = 0.146–0.362)
13406 measured reflections
2664 independent reflections
2315 reflections with *I* > 2σ(*I*)
*R*_int_ = 0.057

#### Refinement

*R*[*F* ^2^ > 2σ(*F* ^2^)] = 0.034
*wR*(*F* ^2^) = 0.087
*S* = 1.09
2664 reflections
181 parameters
H-atom parameters constrained
Δρ_max_ = 3.35 e Å^−3^
Δρ_min_ = −1.31 e Å^−3^

### 

Data collection: *APEX2* (Bruker, 2007 ▶); cell refinement: *SAINT* (Bruker, 2007 ▶); data reduction: *SAINT*; program(s) used to solve structure: *SHELXS97* (Sheldrick, 2008 ▶); program(s) used to refine structure: *SHELXL97* (Sheldrick, 2008 ▶); molecular graphics: *SHELXTL* (Sheldrick, 2008 ▶); software used to prepare material for publication: *SHELXTL*.

## Supplementary Material

Crystal structure: contains datablocks global, I. DOI: 10.1107/S1600536809028542/fj2238sup1.cif

Structure factors: contains datablocks I. DOI: 10.1107/S1600536809028542/fj2238Isup2.hkl

Additional supplementary materials:  crystallographic information; 3D view; checkCIF report

## Figures and Tables

**Table 1:** Selected bond lengths (Å)

Pb1—O3	2.494 (4)
Pb1—O1	2.499 (4)
Pb1—O2	2.515 (5)
Pb1—O5^i^	2.639 (4)
Pb1—O3^ii^	2.677 (4)
Pb1—O5	2.735 (4)
Pb1—C1^ii^	2.867 (6)

Symmetry codes: (i) 
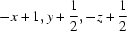
; (ii) 
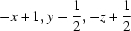
.

**Table 2:** Hydrogen-bond geometry (Å, °)

*D*—H⋯*A*	*D*—H	H⋯*A*	*D*⋯*A*	*D*—H⋯*A*
O5—H5*A*⋯O4^iii^	0.85	1.90	2.734 (7)	168
O5—H5*B*⋯O4	0.85	1.96	2.740 (7)	152

Symmetry code: (iii) 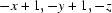.

**Fig. 1.**
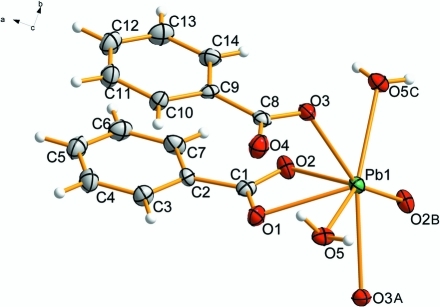
The coordination environment around Pb(II) in the title complex with the atom-labeling scheme. Displacement ellipsoids for non-hydrogen atoms are drawn at the 30% probability level.

**Fig. 2.**
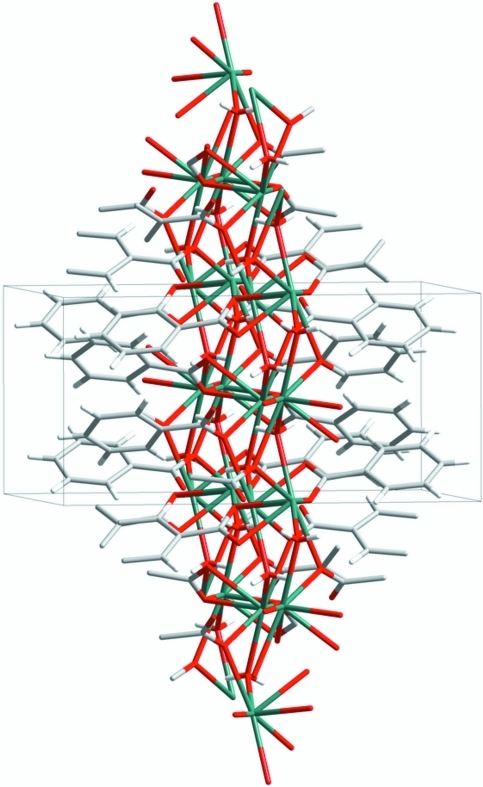
The three-dimensional structure of the title compound.

## supplementary crystallographic information

### Comment

Lead(II) compounds have been increasingly studied (Shi *et al.* 2007; Fan *et al.* 2006) owing to their possible applications in different fields, especially in environmental protection due to the toxicity of lead and in biological systems for its diverse interactions with biological molecules. As an important family of multidentate O-donor ligands, aromatic carboxylate ligands have been extensively employed in the preparation of metal-organic complexes because of their potential properties and intriguing structural topologies (Wang *et al.* 2006; Kim *et al.* 2001). Herein, we report the structure of the title complex.

The asymmetric unit of the title complex, [Pb(C_7_H_5_O_2_)_2_(H_2_O)]_n_, contains a Pb^II^ cation, two BA ligands and one water molecule, as illustrated in Fig. 1. The Pb^II^ atom is heptacoordinated and chelated by two carboxylate O atoms. The Pb atoms are bridged through a coordinating water and two carboxylate O atoms from another two BA ligands. The Pb—O bond lengths are in the range of 2.494 (4) to 2.735 (4) Å. The inter-distance of Pb···Pb is 4.0683 (4) Å, indicating the weak metal-metal interaction. The Pb^II^ atom has a distorted pentagonal bipyramidal geometry and the complexes extend infinitely to three-dimensional supramolecular structure. The coordinating water molecule and carboxylate O atoms are involved in extensive O—H···O hydrogen-bonding interactions (Table 2).

### Experimental

A mixture of Pb(NO_3_)_2_ 3H_2_O (0.172 g, 0.52 mmol), BA (0.102 g, 0.84 mmol), melamine (0.026 g, 0.20 mmol) and distilled water (10 ml) was sealed in a 25 ml Teflon-lined stainless autoclave (Shi *et al.* 2007). The mixture was heated at 403 K for 6 days to give the colorless stick crystals suitable for X-ray diffraction analysis.

### Refinement

All H atoms bounded to C atoms were positioned geometrically and allowed to ride on their parent atoms, with C—H distances of 0.93 Å. The positions of the water H atoms were found from a difference Fourier map and refined with distance restraints O—H = 0.85 Å, *U*_iso_(H) = 1.5Ueq(O).

### Crystal data

**Table:**

[Pb(C_7_H_5_O_2_)_2_(H_2_O)]	*F*(000) = 872
*M**_r_* = 467.43	*D*_x_ = 2.335 Mg m^−^^3^
Monoclinic, *P*2_1_/*c*	Mo *K*α radiation, λ = 0.71073 Å
Hall symbol: -P 2ybc	Cell parameters from 4827 reflections
*a* = 15.4118 (12) Å	θ = 2.6–28.5°
*b* = 7.5122 (6) Å	µ = 12.71 mm^−^^1^
*c* = 11.4856 (9) Å	*T* = 295 K
β = 91.293 (1)°	Block, colorless
*V* = 1329.42 (18) Å^3^	0.4 × 0.1 × 0.08 mm
*Z* = 4	

### Data collection

**Table:**

Bruker APEXII CCD area-detector diffractometer	2664 independent reflections
Radiation source: fine-focus sealed tube	2315 reflections with *I* > 2σ(*I*)
graphite	*R*_int_ = 0.057
φ and ω scans	θ_max_ = 26.2°, θ_min_ = 3.2°
Absorption correction: multi-scan (*SADABS*; Bruker, 2007)	*h* = −19→18
*T*_min_ = 0.1, *T*_max_ = 0.247	*k* = −9→9
13406 measured reflections	*l* = −14→14

### Refinement

**Table:**

Refinement on *F*^2^	Secondary atom site location: difference Fourier map
Least-squares matrix: full	Hydrogen site location: inferred from neighbouring sites
*R*[*F*^2^ > 2σ(*F*^2^)] = 0.034	H-atom parameters constrained
*wR*(*F*^2^) = 0.087	*w* = 1/[σ^2^(*F*_o_^2^) + (0.047*P*)^2^] where *P* = (*F*_o_^2^ + 2*F*_c_^2^)/3
*S* = 1.09	(Δ/σ)_max_ < 0.001
2664 reflections	Δρ_max_ = 3.35 e Å^−^^3^
181 parameters	Δρ_min_ = −1.31 e Å^−^^3^
0 restraints	Extinction correction: *SHELXL97* (Sheldrick, 2008)
Primary atom site location: structure-invariant direct methods	Extinction coefficient: 0.082

### Fractional atomic coordinates and isotropic or equivalent isotropic displacement parameters (Å^2^)

**Table:**

	*x*	*y*	*z*	*U*_iso_*/*U*_eq_	
Pb1	0.478855 (16)	0.52214 (3)	0.311202 (19)	0.02834 (12)	
O1	0.6082 (3)	0.3213 (6)	0.3120 (4)	0.0390 (11)	
O2	0.5947 (4)	0.4879 (5)	0.4674 (4)	0.0401 (12)	
O3	0.5875 (3)	0.6929 (5)	0.1986 (4)	0.0372 (11)	
O4	0.6356 (4)	0.5476 (7)	0.0428 (4)	0.0453 (13)	
O5	0.4960 (3)	0.3455 (6)	0.1048 (3)	0.0349 (10)	
H5A	0.4562	0.3648	0.0539	0.052*	
H5B	0.5374	0.3865	0.0651	0.052*	
C1	0.6397 (4)	0.3861 (8)	0.4045 (5)	0.0310 (14)	
C2	0.7318 (4)	0.3531 (8)	0.4365 (5)	0.0286 (13)	
C3	0.7840 (5)	0.2548 (9)	0.3639 (5)	0.0370 (15)	
H3A	0.7596	0.1994	0.2986	0.044*	
C4	0.8716 (5)	0.2383 (10)	0.3876 (6)	0.0469 (18)	
H4A	0.9062	0.1732	0.3378	0.056*	
C5	0.9082 (5)	0.3177 (10)	0.4847 (6)	0.0495 (19)	
H5C	0.9675	0.3082	0.4996	0.059*	
C6	0.8567 (5)	0.4114 (10)	0.5599 (6)	0.0470 (18)	
H6A	0.8810	0.4624	0.6268	0.056*	
C7	0.7695 (5)	0.4291 (9)	0.5356 (6)	0.0405 (16)	
H7A	0.7351	0.4930	0.5863	0.049*	
C8	0.6480 (4)	0.6270 (8)	0.1387 (5)	0.0309 (14)	
C9	0.7395 (4)	0.6483 (7)	0.1816 (5)	0.0270 (13)	
C10	0.8062 (4)	0.5691 (9)	0.1224 (5)	0.0346 (14)	
H10A	0.7938	0.5033	0.0554	0.042*	
C11	0.8914 (5)	0.5869 (11)	0.1619 (6)	0.0462 (17)	
H11A	0.9361	0.5359	0.1203	0.055*	
C12	0.9098 (5)	0.6794 (11)	0.2623 (6)	0.0503 (19)	
H12A	0.9668	0.6896	0.2898	0.060*	
C13	0.8433 (5)	0.7573 (10)	0.3222 (6)	0.050 (2)	
H13A	0.8560	0.8209	0.3899	0.060*	
C14	0.7587 (5)	0.7427 (9)	0.2838 (6)	0.0408 (17)	
H14A	0.7144	0.7952	0.3254	0.049*	

### Atomic displacement parameters (Å^2^)

**Table:**

	*U*^11^	*U*^22^	*U*^33^	*U*^12^	*U*^13^	*U*^23^
Pb1	0.02962 (19)	0.02363 (16)	0.03171 (17)	0.00173 (9)	−0.00073 (11)	0.00129 (8)
O1	0.030 (3)	0.042 (3)	0.044 (3)	0.001 (2)	−0.010 (2)	−0.004 (2)
O2	0.039 (3)	0.041 (3)	0.041 (3)	0.015 (2)	0.005 (2)	0.0009 (19)
O3	0.036 (3)	0.027 (2)	0.049 (3)	0.0032 (19)	0.011 (2)	0.0011 (19)
O4	0.037 (3)	0.056 (3)	0.043 (3)	−0.001 (2)	−0.004 (2)	−0.006 (2)
O5	0.043 (3)	0.033 (2)	0.028 (2)	−0.005 (2)	−0.0034 (19)	0.0054 (17)
C1	0.035 (4)	0.030 (3)	0.028 (3)	−0.003 (3)	−0.001 (3)	0.009 (2)
C2	0.027 (4)	0.026 (3)	0.032 (3)	0.004 (2)	−0.002 (3)	0.003 (2)
C3	0.039 (4)	0.041 (4)	0.031 (3)	0.001 (3)	−0.005 (3)	−0.003 (3)
C4	0.037 (5)	0.053 (5)	0.051 (4)	0.005 (3)	0.008 (3)	0.003 (3)
C5	0.034 (4)	0.057 (5)	0.057 (5)	−0.003 (4)	−0.006 (4)	0.012 (4)
C6	0.052 (5)	0.045 (4)	0.044 (4)	−0.006 (4)	−0.011 (4)	−0.002 (3)
C7	0.048 (5)	0.038 (4)	0.035 (4)	0.005 (3)	−0.010 (3)	−0.008 (3)
C8	0.037 (4)	0.021 (3)	0.035 (3)	0.002 (3)	0.001 (3)	0.006 (2)
C9	0.031 (4)	0.022 (3)	0.028 (3)	−0.006 (2)	0.000 (2)	0.006 (2)
C10	0.029 (4)	0.041 (4)	0.034 (3)	−0.003 (3)	0.002 (3)	−0.003 (3)
C11	0.033 (4)	0.055 (5)	0.051 (4)	0.003 (4)	0.006 (3)	−0.004 (4)
C12	0.039 (5)	0.060 (5)	0.052 (4)	−0.004 (4)	−0.012 (4)	0.002 (4)
C13	0.057 (6)	0.053 (5)	0.039 (4)	−0.008 (4)	−0.012 (4)	−0.007 (3)
C14	0.048 (5)	0.033 (4)	0.041 (4)	−0.004 (3)	0.008 (3)	−0.005 (3)

### Geometric parameters (Å, °)

**Table:**

Pb1—O3	2.494 (4)	C4—C5	1.374 (10)
Pb1—O1	2.499 (4)	C4—H4A	0.9300
Pb1—O2	2.515 (5)	C5—C6	1.379 (10)
Pb1—O5^i^	2.639 (4)	C5—H5C	0.9300
Pb1—O3^ii^	2.677 (4)	C6—C7	1.374 (10)
Pb1—O5	2.735 (4)	C6—H6A	0.9300
Pb1—C1	2.867 (6)	C7—H7A	0.9300
O1—C1	1.256 (7)	C8—C9	1.493 (8)
O2—C1	1.269 (8)	C9—C10	1.379 (9)
O3—C8	1.271 (7)	C9—C14	1.397 (8)
O3—Pb1^i^	2.677 (4)	C10—C11	1.386 (10)
O4—C8	1.264 (7)	C10—H10A	0.9300
O5—Pb1^ii^	2.639 (4)	C11—C12	1.370 (10)
O5—H5A	0.8500	C11—H11A	0.9300
O5—H5B	0.8500	C12—C13	1.378 (10)
C1—C2	1.479 (8)	C12—H12A	0.9300
C2—C3	1.385 (8)	C13—C14	1.371 (10)
C2—C7	1.389 (8)	C13—H13A	0.9300
C3—C4	1.377 (10)	C14—H14A	0.9300
C3—H3A	0.9300		
			
O3—Pb1—O1	76.58 (15)	C3—C2—C1	120.5 (5)
O3—Pb1—O2	87.06 (16)	C7—C2—C1	121.1 (6)
O1—Pb1—O2	51.87 (14)	C4—C3—C2	120.6 (6)
O3—Pb1—O5^i^	67.76 (13)	C4—C3—H3A	119.7
O1—Pb1—O5^i^	116.34 (14)	C2—C3—H3A	119.7
O2—Pb1—O5^i^	74.78 (13)	C5—C4—C3	120.2 (7)
O3—Pb1—O3^ii^	135.53 (10)	C5—C4—H4A	119.9
O1—Pb1—O3^ii^	75.30 (14)	C3—C4—H4A	119.9
O2—Pb1—O3^ii^	101.51 (14)	C4—C5—C6	119.9 (7)
O5^i^—Pb1—O3^ii^	156.67 (12)	C4—C5—H5C	120.0
O3—Pb1—O5	73.78 (13)	C6—C5—H5C	120.0
O1—Pb1—O5	67.52 (13)	C7—C6—C5	119.7 (7)
O2—Pb1—O5	119.18 (14)	C7—C6—H6A	120.1
O5^i^—Pb1—O5	138.36 (11)	C5—C6—H6A	120.1
O3^ii^—Pb1—O5	63.89 (12)	C6—C7—C2	121.1 (7)
O3—Pb1—C1	78.02 (15)	C6—C7—H7A	119.5
O1—Pb1—C1	25.93 (15)	C2—C7—H7A	119.5
O2—Pb1—C1	26.25 (15)	O4—C8—O3	123.8 (6)
O5^i^—Pb1—C1	94.18 (15)	O4—C8—C9	117.5 (6)
O3^ii^—Pb1—C1	90.71 (16)	O3—C8—C9	118.7 (5)
O5—Pb1—C1	92.94 (15)	C10—C9—C14	119.2 (6)
C1—O1—Pb1	93.6 (4)	C10—C9—C8	120.0 (5)
C1—O2—Pb1	92.5 (4)	C14—C9—C8	120.8 (6)
C8—O3—Pb1	126.1 (4)	C9—C10—C11	120.5 (6)
C8—O3—Pb1^i^	128.4 (4)	C9—C10—H10A	119.8
Pb1—O3—Pb1^i^	103.70 (15)	C11—C10—H10A	119.8
Pb1^ii^—O5—Pb1	98.38 (12)	C12—C11—C10	120.1 (7)
Pb1^ii^—O5—H5A	120.4	C12—C11—H11A	119.9
Pb1—O5—H5A	115.5	C10—C11—H11A	119.9
Pb1^ii^—O5—H5B	115.0	C11—C12—C13	119.5 (7)
Pb1—O5—H5B	112.2	C11—C12—H12A	120.3
H5A—O5—H5B	96.3	C13—C12—H12A	120.3
O1—C1—O2	120.6 (6)	C14—C13—C12	121.2 (7)
O1—C1—C2	119.7 (6)	C14—C13—H13A	119.4
O2—C1—C2	119.5 (6)	C12—C13—H13A	119.4
O1—C1—Pb1	60.5 (3)	C13—C14—C9	119.5 (7)
O2—C1—Pb1	61.2 (4)	C13—C14—H14A	120.3
C2—C1—Pb1	165.8 (4)	C9—C14—H14A	120.3
C3—C2—C7	118.3 (6)		

Symmetry codes: (i) −*x*+1, *y*+1/2, −*z*+1/2; (ii) −*x*+1, *y*−1/2, −*z*+1/2.

### Hydrogen-bond geometry (Å, °)

**Table:**

*D*—H···*A*	*D*—H	H···*A*	*D*···*A*	*D*—H···*A*
O5—H5A···O4^iii^	0.85	1.90	2.734 (7)	168
O5—H5B···O4	0.85	1.96	2.740 (7)	152

Symmetry codes: (iii) −*x*+1, −*y*+1, −*z*.
